# Analysis of Failure Mechanism in Joints with Positive Eccentricity in CFS Truss

**DOI:** 10.3390/ma14226986

**Published:** 2021-11-18

**Authors:** Małgorzata Gordziej-Zagórowska, Elżbieta Urbańska-Galewska, Patryk Deniziak

**Affiliations:** Faculty of Civil and Environmental Engineering, Gdansk University of Technology, 80-233 Gdansk, Poland; ugalew@pg.edu.pl (E.U.-G.); patdeniz@pg.edu.pl (P.D.)

**Keywords:** stability, truss, eccentricities in the truss joints, numerical investigation

## Abstract

The paper presents studies concerning the load-bearing capacity of truss joints with a positive eccentricity resulting from the arrangement of geometric members and the failure mechanisms observed in the joints. Based on the previously conducted experimental study, a numerical model of the tested fragment of the CFS truss with eccentricity in the joint was created and validated. All structural details of the tested truss and the loading method consistent with the experiment were taken into account. The results obtained from a uniaxial tensile tests on a steel samples and results estimated during destructive tests carried out on a full-scale of research model were taken into account in validation of the numerical model. Next, appropriate numerical analyses were carried out and parameters such as the eccentricity size in the joint and the wall thickness of sections (*t* = 1.0, 1.5 and 4.0 mm) were modified. In the range of the studied wall slenderness from λ > 70 (1.0 mm and 1.5 mm thick), it was confirmed that the resistance of truss joints made from CFS open cross-sections with a positive eccentricity, is greater than the resistance that results from known methods of steel structure dimensioning.

## 1. Introduction

Truss girders made of cold-formed steel (CFS) members are designed in accordance with European standards [1,2,3,4]. However, rules of the afore-mentioned standards do not take into account the specificity of the construction of truss joints made from open cross-section profiles with a positive eccentricity in the truss joints. The presence of an eccentricity increases the level of cross-section utilization of the truss chords in the joint area. This either leads to an increased cross-section of the whole chord or requires additional elements in the joint in order to increase the local stiffness of the element wall. Therefore, experimental and numerical studies were carried out to check the actual state of stress and strain in the eccentric joint of truss girders made from open cross-sections.

**Figure 1:**
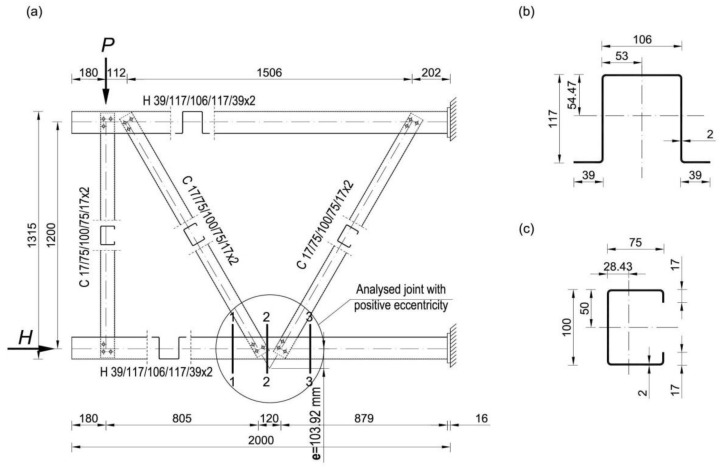
(**a**) Research model; static scheme, dimensions of sections used in experimental tests for: (**b**) chords, and (**c**) diagonals (All dimensions in mm).

Figure 1 presents the dimensions of the research model with the location of the measurement sections in the area of the analysed joint. The truss was made of CFS open cross-sections made of S350GD steel. Chords were made from the hat-section H 39/117/106/117/39x2, while diagonals were made from the channel-section C 17/75/100/75/17x2 (Figure 1b,c). The connections in each joint were made with 6 bolts M12, class 8.8 (3 bolts in each web).

The experimental tests presented in [5] were the first stage of the research aimed at obtaining information about the behaviour of the truss with positive eccentricities. The research also aimed to obtain results in the form of a registered strains and displacements of the model, and joint deformations. A detailed description of the research setup and the reason for undertaking the research, together with the details of the experimental tests and resulting conclusions, are presented in the paper [5]. Due to the high costs of experimental research, the study was limited to a scrupulously designed model of the truss section with only one wall thickness. A series of destructive tests was carried out on five models and a high degree of convergence was achieved. However, the obtained results did not allow us to confirm the research hypothesis at this stage but were the basis for validation of the numerical model. Furthermore, it was found that the calculations of the analysed section of the truss carried out in accordance with the Eurocode procedures [1,2,3,4] corresponded to the experimental results for the truss made of 2 mm thick profiles. This publication is the second stage of the research on CFS trusses with eccentric joints. The article presents the results of a series of numerical analyses for variable wall thicknesses of steel profiles (*t* = 1.0, 1.5 and 4.0 mm) and different eccentricity values in the joint. Increasing the scope of the analysed truss variants made it possible to verify the research hypothesis put forward in [5].

## 2. State of the Art

Due to the above-mentioned limitations and high costs of experimental research, many scientific studies on the resistance and stability of complex structural elements are limited to the analysis of numerical models using various method [6,7]. In their publication [6] Camotim and Basaglia presented a comprehensive report on their research using the Generalized Beam Theory (GBT) at the buckling analysis. The GBT-based beam finite element method allowed for analysis of various forms of local, distortional and global stability loss, both of individual thin-walled members and structures made from them. One of the structures analysed was a section of a truss made from cold-formed channel sections applied to chords and diagonals (research also described in detail in Basaglia and Camotim [8]). The connections in the truss joints are made by using a pair of bolts located at the intersection of the axes of the diagonals and the chords. Two variants of trusses (short and tall) were used for the analysis. The aim of the study was to numerically analyse the local, distortional and global buckling of truss members made of thin-walled sections using the GBT method. The GBT model was validated using ANSYS numerical software. The analyses carried out allowed the critical load to be determined and the forms of deformation of the truss members to be identified. For a short truss, the local form of loss of stability of the lower chord in the section between joints, and for a tall truss, the global buckling (flexural–torsional) of both diagonals, were proved to be critical. The results were found to be satisfactory using the GBT method and MES analysis.

In turn, Visy, Adany and Joo [7] conducted a global buckling analysis of a triangular truss made of thin-walled cold-formed hat sections with edge stiffeners used for chords and diagonals. The research was carried out for several variants of dimensions and loads. The hat sections of the chords were situated with the open part facing the centre of the truss, as a result of which the diagonals could be inserted into the chords. Chords were connected with diagonals by only one bolt (common for both walls of the connected hat sections). The main objective of the study was to investigate the effects of flexural–torsional buckling and lateral–torsional buckling of the truss members on the values of the internal forces and critical moments. The effect of side transverse bracing of the upper chord was also investigated.

Eurocodes allow numerical (FEA) solutions for design issues provided that the models have been validated by appropriate experimental testing. This approach is presented in the latest cold-formed section structural studies, which consist of two stages: experimental and numerical. Thus, Pham and Hancock have developed their studies on the effect of the web’s longitudinal stiffeners on the shear stability of the channel web by a number of numerical analyses [9,10]. Crisan, Ungureanu and Dubina conducted buckling behaviour tests of storage rack members made of sigma-type cross-section (with and without perforation). At first a single upright section was experimentally and numerically tested [11,12]. Next the full-scale stability tests of frame modules was conducted [13]. Based on research results for both single upright sections and frame modules authors demonstrate that the design buckling strength of uprights can be conveniently estimated by single section tests, providing that the length of these members is calibrated for the distortional–global interaction mode.

Much attention is paid to lattice roof girders made of CFS connected in joints with self-drilling screws [14], self-piercing rivets (SPR) [15] or a novel pin-jointed moment connector, called the Howick Rivet Connector (HRC) presented by Mathieson at al. [16,17]. Song, Yan, Yo, Xie and Tan [15] carried out an experimental study of five trusses made of CFS sections connected by self-piercing rivets (SPR). The trusses were bent at one point. The effect of the height and span of the truss on its stiffness and bending resistance was investigated. After the experimental tests, numerical FEM analyses of the tested models were performed, taking into account additional parameters. As a result of the analyses, a theoretical formula was proposed to calculate the equivalent bending stiffness of a lattice beam with SPR connections on the basis of parametric analysis. The formula agreed with the results of the experiment, so it was considered that it could be used in engineering practice.

Dizdar, Baran and Topkaya [18] conducted experimental and numerical research on 17 full-scale floor trusses made of cold-formed sigma-type cross-sections with edge stiffeners. The connections in the truss were made with rivets or self-tapping screws. A numerical model of the tested truss was developed in order to carry out buckling analyses, changing parameters such as the thickness of the chord wall and the shape of the cross-section (channel with and without edge stiffeners).

Bondok, Salim and Urgessa [19] conducted cold-formed steel roof truss segments research due to failure under quasi-static loading. The experimental tests were carried out on five small-scale samples. Experimental results and finite element analyses showed that the truss layout and the shape of loading significantly affected the performance of the truss and the failure mechanism. When the applied load was localized at a single point, the truss stiffness and the ultimate capacity decreased. While the web members susceptible to buckling were strengthened the absorbed energy was significantly improved. In future studies, the authors would like to use a verified finite element model to predict the resistance and the energy absorption capacity of full-scale trusses.

In the latest publications on CFS trusses a new direction of research can be noticed. Due to the stability loss of thin-walled profiles used for truss members, scientists proposed to infilled concrete in selected truss members. Guldur, Baran and Topkaya [20] presented experimental and numerical study of CFS floor trusses made of lipped channel sections with the compression chord member filled with concrete. At first seven full-scale CFS floor trusses were experimentally tested. Then numerical investigation included three-dimensional finite element modelling of these trusses was performed. Test results showed that presence of concrete infill inside the compression chord member prevented the initiation of chord local/distortional buckling, which obtained in a significant increase in the stiffness and load capacity of the truss. Ma, Liu, Wang, Liu and Zhang [21] conducted numerical investigation of the concrete filled rectangular steel tubular (CFRST) truss joints. The effects were different for the brace under tension and compression. The calculated equations of the axial stiffness of CFRST joints (T, Y and K) were adopted to predict the flexural stiffness of CFRST trusses.

In summary, few studies on cold-formed lattice girders relate to open sections. Currently, for reasons of fast structure execution, scientists are looking for solutions to efficiently connect truss members in joints using fasteners such as self-drilling screws or self-piercing rivets. However, so far, no studies have been undertaken to determine the effect of the eccentricity on the stability of the chord walls in a truss joint made of cold-formed open cross-sections.

## 3. Calibration and Validation of the Numerical Model

### 3.1. Numerical Model

**Figure 2:**
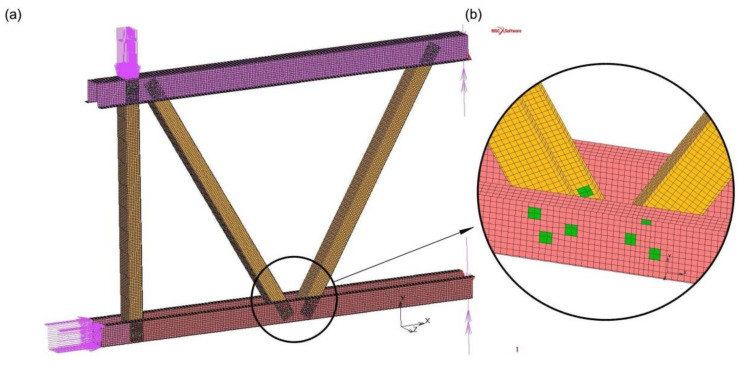
Geometry of the computational model (load, supports): (**a**) truss model, (**b**) the analysed joint.

**Figure 3:**
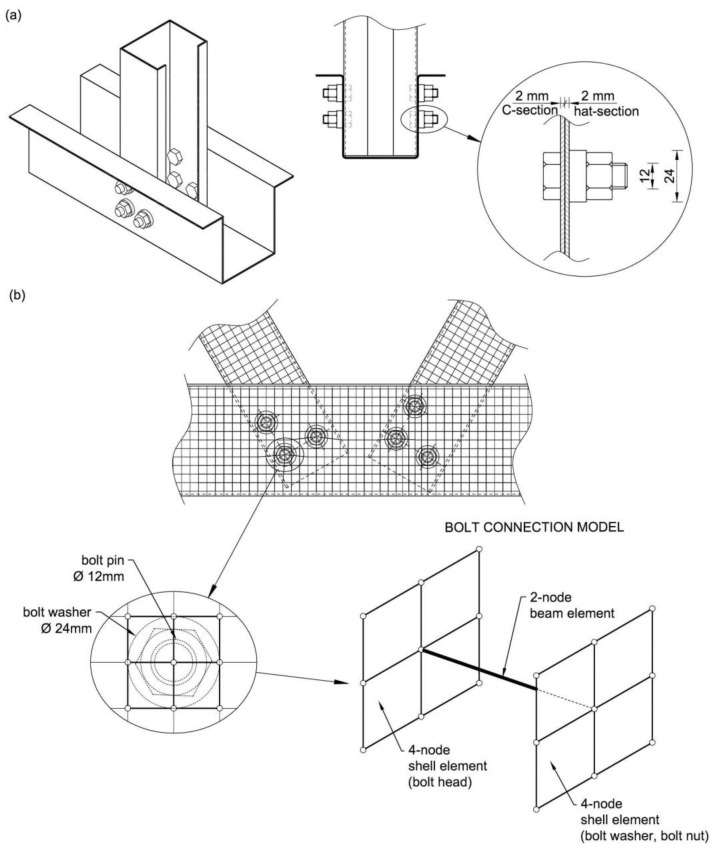
Bolt connection: (**a**) real, (**b**) computational model.

The computational model of the experimentally tested truss segment [5] was performed in MSC Marc software (2013.0.0) using the finite element method (FEM). Figure 2 shows the geometry of the computational model implemented in MSC Marc. The model was made using shell elements. The size and type of grid elements, and the way of division into finite elements, were tested in order to obtain sufficient accuracy of the results at the optimum computing time. Finally, four-node rectangular shell elements of about 8 × 8 mm dimensions were used to model thin-walled sections. The bolted connection model was made from two-node beam elements and four-node shell elements in accordance with the scheme shown in Figure 3.

In the real tested structure, the walls of the channel section and hat sections with a thickness of 2 mm each are connected by prestressed M12 bolts, grade 8.8, washers with a diameter of 24 mm, and nuts. Therefore, the fastener consisting of a bolt, a washer and a nut provides additional local stiffening of the two adjacent section walls, each 2 mm thick (Figure 3a). In addition, experimental studies [5] have shown that the prestressing of the bolted connections used in the assembly of the truss ensures that the surfaces of the connected members adhere sufficiently. This prevents the edges of a bolt hole from being damaged by the bearing of the bolt pin. Considering the above, it was decided to model the bolted connection as a point-connected, stiffened wall zone of the channel and hats, as in the real truss.

The prepared bolt connection model consists of a two-node beam element imitating the bolt pin, and two rigid zones each consisting of four four-node shell elements (Figure 3b) imitating the bolt head on one side and the washer and nut on the other. The dimensions of the shell elements were compatible with the real size of the washer/nut, as shown in Figure 3b.

In the MSC Marc software, the two-node beam element was given geometric characteristics of the M12 bolt shank (cross-sectional area and inertia moments), while the material was defined as linear with Young’s modulus E = 210 GPa. The shell elements in the bolted connection model were also defined with a material with a linear characteristic but with much higher stiffness (both the thickness of the shell and Young’s modulus of elasticity were increased). As a result, local stiffness was obtained in the area of the bolted connection, which in the real model is realised by means of washers, nuts or bolt heads. This model was considered sufficient at this stage of the study because the main objective of this research was to analyse the behaviour of the thin-walled members’ walls in the area of the joint, not the bolts themselves. Bolt connection models were situated at the real bolt locations, which provided a point connection between the channel-section diagonals and the hat-section chords.

### 3.2. Boundary Conditions and Applied Load

**Figure 4:**
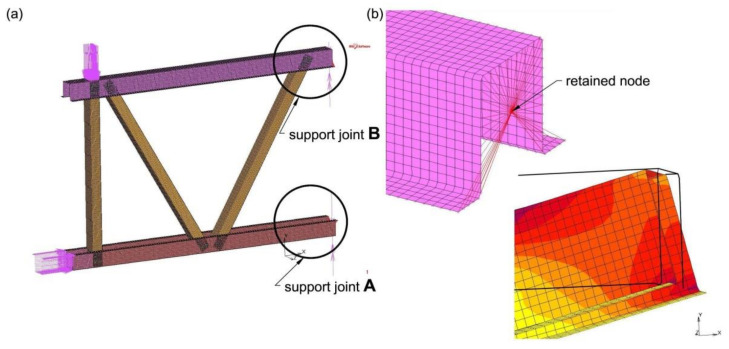
Model supports-RBE2 element: (**a**) view of the model, (**b**) detail of the support joint.

The support points of the computational model were placed in the centroid of the hat section using the RBE2 element (nodal tie). This element consists of one “retained node”, located in the centroid of the hat section, for the top and bottom chord respectively, and for any number of “tied nodes” (Figure 4). The nature of the relationship between the retained node and tied nodes is rigid, which means that assigned nodes are rigidly connected to the retained node (all degrees of freedom are locked).

**Figure 5:**
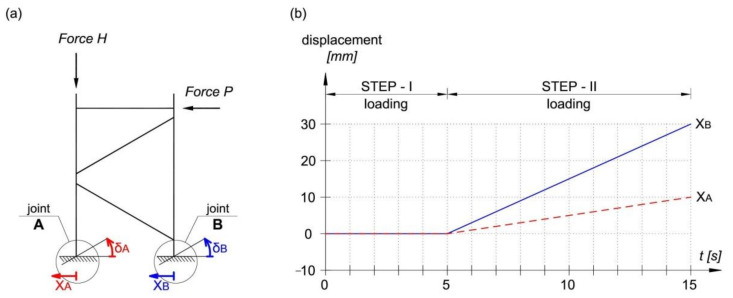
Displacement of support joints: (**a**) model view, (**b**) displacement characteristic dependent on time.

At the stage of designing the research model, fixed supports were used. However, the support displacement control (joints A and B) carried out during the experimental tests showed minimal rotation and displacement of the supports, as shown in Figure 5. Therefore, in order to verify the correctness of the validation model, it was necessary to imitate the support joints A and B in the MSC Marc software which was exactly the same as in the experimental tests. Pin supports with the displacement characteristic shown in Figure 5b were used.

**Figure A1:**
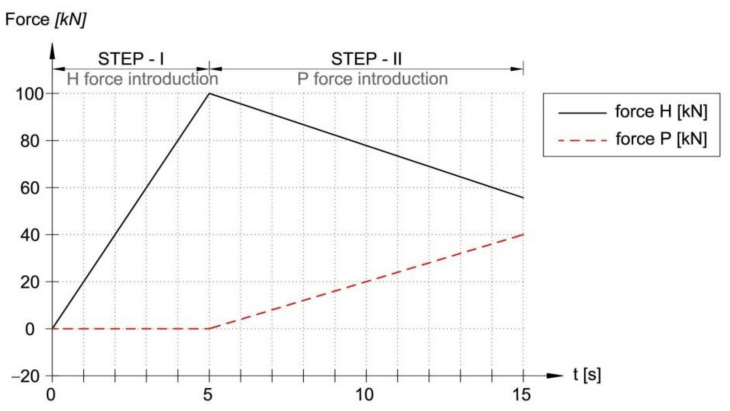
A simplified graph of the load history vs. time applied in numerical analysis.

The *P* and *H* loads for the computational model were applied via nodes. Force *H* was applied to each node located on the cross-section contour (Figure 4). However, force *P* was applied to the selected web surface of the top chord (Figure 4), which represented the real introduction of force in the experiment. The analyses also included the research model’s own weight. Forces *H* and *P* were applied to the validation model according to the load history recorded during destructive tests. A simplified graph of the load history during the analysis time is shown in Figure A1.

### 3.3. Parameters of Nonlinear Analysis and Material Model

Geometrically and materially nonlinear analysis (GMNA) was used during numerical research. For the purposes of model validation, the material characteristics of steel used during experimental tests (elastic-plastic material model) were introduced. The following parameters were used: hat-section *E* = 210 GPa, *f_y_* = 398 MPa, *f_u_* = 489 MPa, channel-section *E* = 199 GPa, *f_y_* = 366 MPa, *f_u_* = 445 MPa. The relation true stress–true strain for both hat and channel sections was applied. Material, technological and geometrical imperfections were omitted during the analysis.

During the validation analysis, the iterative Newton–Raphson method was used to solve the nonlinear problem [22]. In order to select the size of the time steps, the adaptive procedure-multi-criteria was chosen. In the multi-criteria procedure, the time step of the analysis is automatically selected using the damping energy criterion or the iterative criterion. This way of solving the non-linear problem allowed for a gradual introduction of a load consisting of two forces: H and P, which were applied to the model one after the other. While performing appropriate numerical analyses, forces H and P were introduced to the model at the same time (Figure A3). This allowed the arc-length method to be employed, making use of the modified Riks-Ramm variant [22].

### 3.4. Comparison of the Results of Experimental Studies with the Validation Analysis

**Figure 6:**
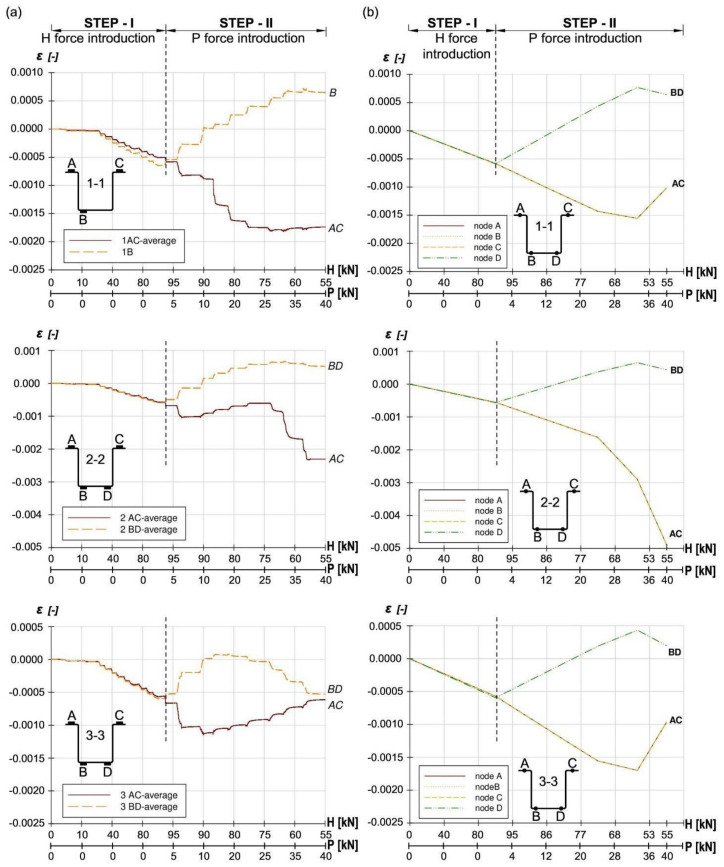
Comparison of strain in three measurement cross-sections: (1-1, 2-2 and 3-3): (**a**) experimental tests, (**b**) numerical analysis, (description in the text).

**Figure 7:**
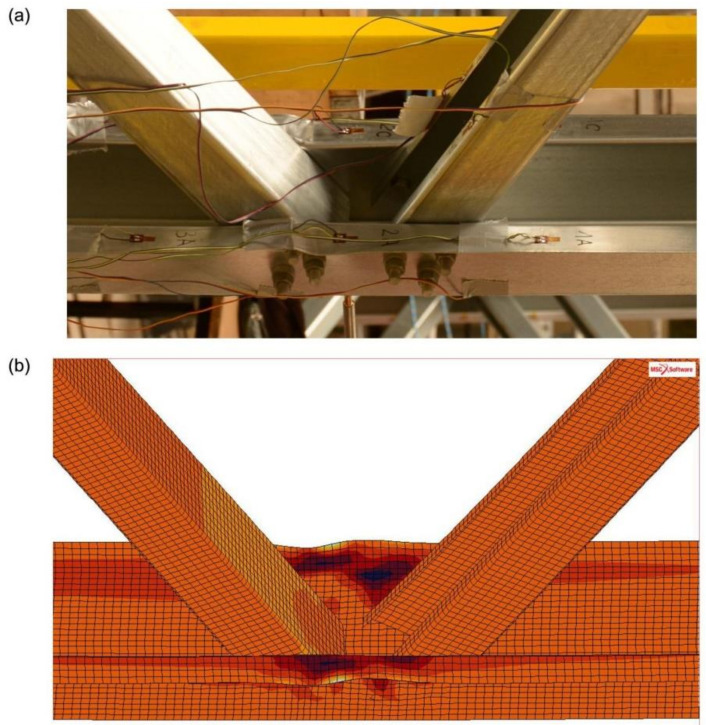
Comparison of failure mode of the analysed joint: (**a**) experimental tests (model no. 4), (**b**) numerical analysis.

Comparison of experimental research results with validation analysis was made on the basis of measured strains in sections 1-1, 2-2 and 3-3 (Figure 1), and comparison of the failure mode of the analysed joint. In order to compare the experimental results with the validation analysis, the values of strain gauge B and D, as well as A and C, were calculated as mean values (marked as “AC” and “BD”), which partially compensated for the effects of imperfections in experimental models. Figure 6a presents the results of strains from experimental tests, presented as mean values of the “AC” and “BD” strain gauges in measuring sections 1-1, 2-2 and 3-3. However, Figure 6b shows the strain values obtained from the numerical analysis of the finite element method at chosen nodes (node A, B, C and D-the location of the nodes corresponds to the location of strain gauges from experimental tests) for the same measuring sections, 1-1, 2-2 and 3-3 respectively. Figure 7 presents a comparison of the failure mode of the analysed joint for the final stage of load, obtained on the basis of experimental and numerical tests.

The destructive tests carried out on five models of trusses made it possible to determine the level of strains and displacements of the tested structure at selected points and sections. The data received was used to verify the correctness of the build-up numerical model. Comparison of strain results and failure modes shows that the results of numerical analysis are similar to the results of tests on the real model with sufficient accuracy. Due to the high costs of experimental research, it was not possible to perform tests for different truss variants (wall thickness, eccentricity value). It is also impossible to draw general conclusions based on the experimental research of only one specific case. Therefore, the validation of the computational model made it possible to verify the correctness of the numerical model performed. It was necessary to conduct more numerical analyses of different cases to determine the influence of varied values of eccentricity on the load capacity of the truss joint.

## 4. Numerical Analyses

Validation of the computational model made it possible to check that the numerical model correctly describes the real behaviour of the experimentally tested structure. When the validation of the computational model was completed, appropriate numerical analyses were carried out, with modified parameters such as the eccentricity value or wall thickness of the members from which the models were made.

### 4.1. Variants of Numerical Analyses

**Figure 8:**
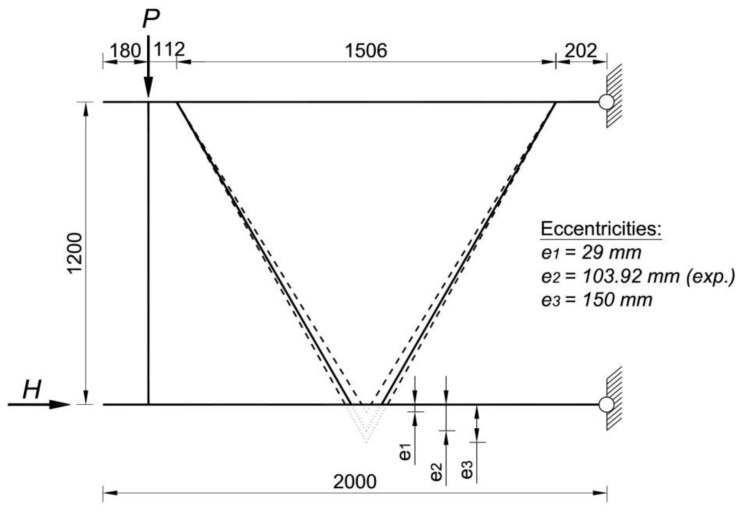
Static scheme of the numerical model with variants of the tested eccentricity values (all dimensions in [mm]).

The developed numerical model allowed the research scope to be extended by modification of selected parameters such as model supports, material model, load history, and geometrical parameters: wall thickness of the section and eccentricity value. The pinned supports of the model were introduced in accordance with the static scheme presented in Figure 8.

**Figure A2:**
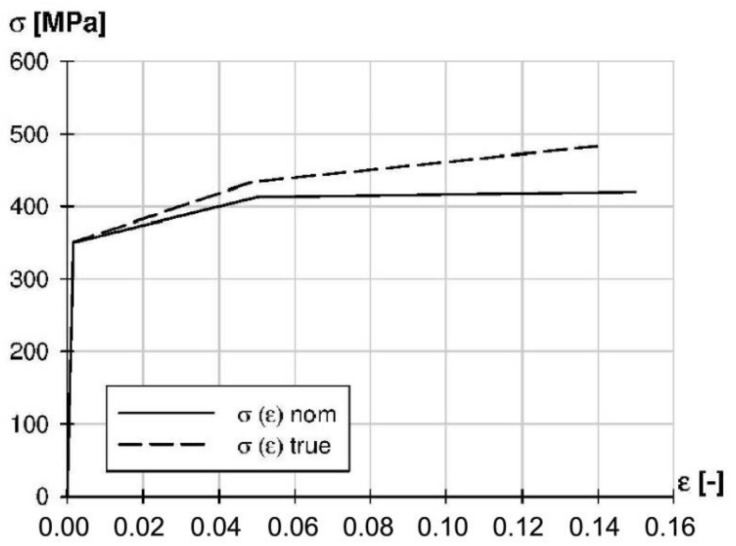
The σ-ε relationship diagram for S350GD steel, used for appropriate numerical analyses.

**Figure A3:**
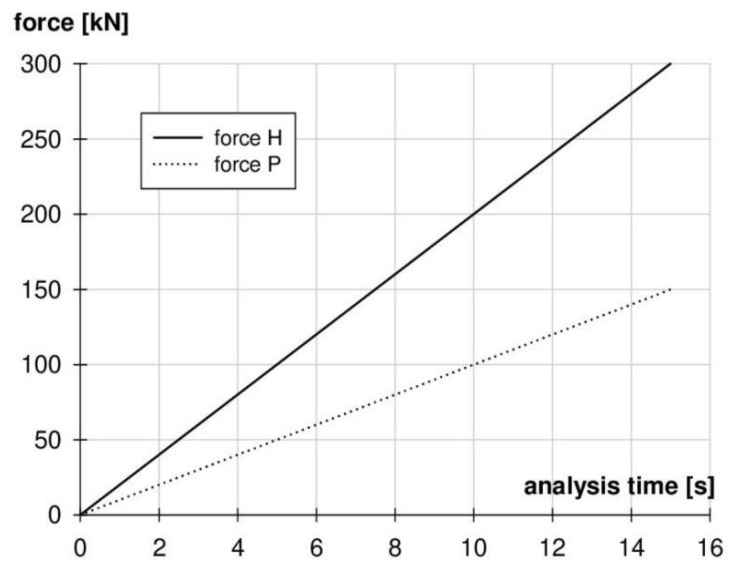
Load history used for appropriate numerical analyses.

The numerical models used the elastic-plastic material model with reinforcement shown in Figure A2. Due to the fact that the purpose of the research was to determine the general relationship regarding the influence of the eccentricity value on the load capacity of the truss joint, the adopted material data had to correspond to the characteristics specified in the standards. For this purpose, the parameters of S350GD steel given in EN 1993-1-1 [1] were used: *f_y_* = 350 MPa, *f_u_* = 420 MPa, *E* = 210 GPa. Load *H* and *P* were introduced simultaneously in the proportion 2:1, in accordance with the diagram in Figure A3. For the above-mentioned parameters, numerical tests were carried out also modifying such geometrical parameters as eccentricity value and wall thickness of truss sections, in accordance with the listing presented in Table 1.

**Figure 9:**
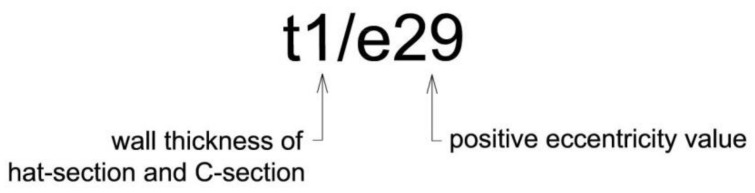
Symbolic name of different variants of numerical analysis.

**Figure 10:**
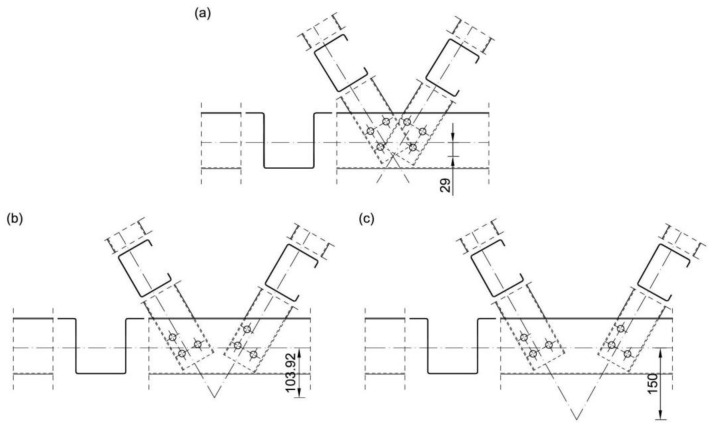
Details of the numerically analysed eccentric joint: (**a**) *e*_1_ = 29 mm, (**b**) *e*_2_ = 103.92 mm, (**c**) *e*_3_ = 150 mm.

Numerical analyses were carried out for four different wall thicknesses of sections: 1 mm, 1.5 mm, 2 mm and 4 mm. The wall thickness in each model was the same for the channel and hat sections. For each variant of wall thickness three different values of positive eccentricity were taken into account: *e*_1_ = 29 mm (“small” eccentricity), *e*_2_ = 103.92 mm (“typical” eccentricity) and *e*_3_ = 150 mm (“large” eccentricity). Figure 9 presents a description of the symbolic name of different variants of numerical analysis used in the research, whereas Figure 10 presents the construction of the analysed joint, taking into account three variants of the eccentric value ‘*e*’.

The eccentricity e_2_ corresponds to the research case of a typical truss joint solution used in the hall system of an international company. The eccentricities *e*_1_ and *e*_3_ are selected as smaller and larger eccentricities respectively, relative to the eccentricity e_2_. The eccentricity values *e*_1_ = 29 mm and *e*_3_ = 150 mm were assumed to be small and large respectively by analogy to eccentricities in nodes from closed sections EN 1993-1-8 [4].

It should be underlined that the eccentricity e_1_ in the case of trusses consisting of open cross-section profiles with bolted connection in joints is a typical example of a purely theoretical solution. In real, such situation is not possible due to the collision between diagonals in truss joint. On the other hand, for analysis purposes the eccentricity e_3_ was chosen so that it was significantly larger than the eccentricity *e*_2_.

### 4.2. Results of the Numerical Analyses

**Figure 11:**
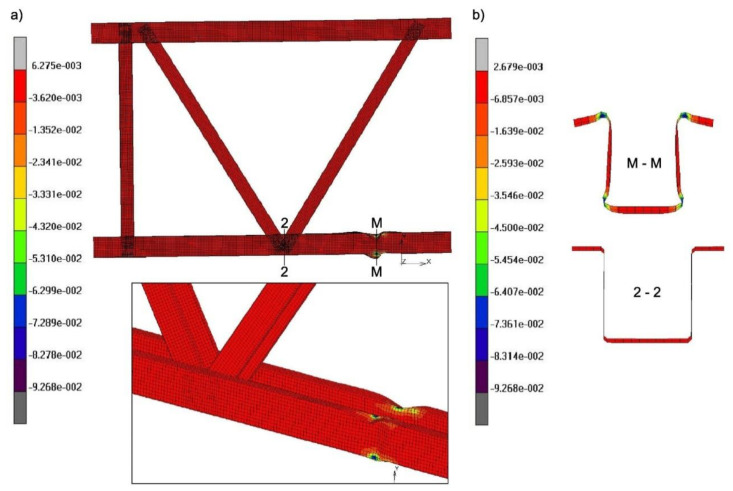
Model t1.5/e29–the failure modes with the strain maps of: (**a**) the whole model and the analysed joint, (**b**) 2-2 and M-M cross-sections.

The purpose of each analysis was to investigate the failure mechanism by determining:

1. The strain state in the cross-section situated in the axis of symmetry of the analysed joint with the eccentricity, marked as cross-section 2-2 (Figure 11),
2. The strain state in section M-M, defined as the cross-section in which the greatest deformations occurred (Figure 11). This can take place between and outside the diagonals,
3. The ultimate limit state of the analysed truss model.

The critical load was obtained from linear elastic bifurcation analysis (LBA) for a group of P and H forces. The ultimate load was determined from geometrically and materially nonlinear analysis (GMNA) using the arc-length method (modified Riks-Ramm).

**Figure 12:**
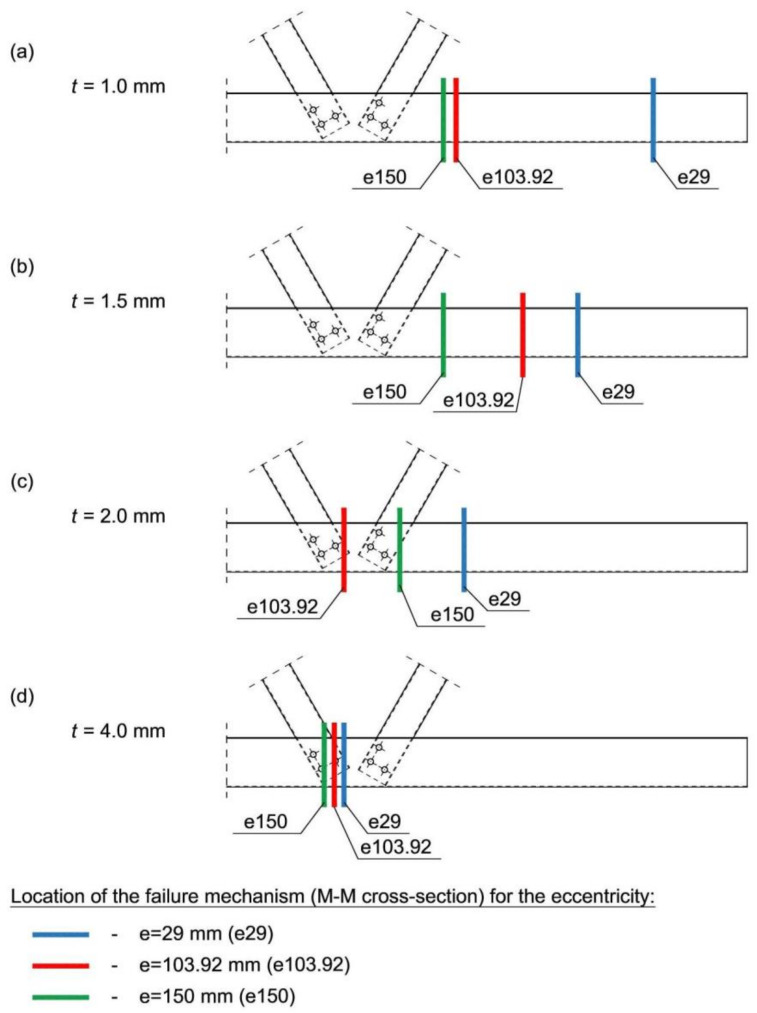
Location of the failure as presented for the M-M cross-section for all variants of numerical analyses.

Based on the analysis of the results for the models: t1/e29; t1/e103.92; t1/e150; t1.5/e29; t1.5/e130.92; t1.5/e150 and t2/e29 it was possible to differentiate between three states that were observed: critical, ultimate and damage. Critical state in each case was characterised by local loss of stability of the compressed chord made of a hat section that occurred in any localisation from the analysed node to the model support. The ultimate state depends on the deformation of the members until the yield point at the corners of the most heavily stressed section of the truss element is reached. Typical failure mechanisms were also observed in compressed open cross-section elements: CF1 consisting in the formation of plastic bends on the chord flanges supported along one edge, and CW1 consisting in the formation of plastic bends on the chord flanges supported along two edges (flip disc type) [23]). In all the above-mentioned cases, the ultimate load was always higher than the critical load (Table 2), which is consistent with the theory of supercritical load-bearing capacity of panels. Most importantly, no effect of shear from bending on the resistance of the joint was found, as the damage, that is shown in section M-M (according to Figure 11), which is critical to the state of failure of the section, always occurred outside the joint, regardless of the eccentricity value (in the compressed part of the chord-Figure 12).

In the case of models t2/e103.92 and t2/e150, the damage of the M-M cross-section occurred in the area of the bend joint; however, this time no shear effect was observed. For the eccentricity equal to *e* = 150, the previously discussed mechanism of destruction (CW1) occurred [23].

The mechanisms of destruction in a truss made from Class 2 cross-sections (models: t4/e29, t4/e103.92 and t4/e150) have a completely different character. In all analysed variants, the damage of the M-M cross-section, critical for the destruction of the section of the compressed chord of the truss, is the cross-section situated between the diagonals of the analysed joint (Figure 12d). This cross-section becomes plasticised during compression and bending. A significant shear effect is visible in the form of plasticising the web zone of the hat section. The value of the critical load corresponded to the global buckling mode and was, in each case, greater than the value of the ultimate load (Table 2), which is consistent with the theory of the limit resistance of compression members.

For all model variants with wall thicknesses of 1 mm, 1.5 mm and 2 mm (Class 4 sections) it can be noted that in cross-section 2-2 the hat section stays within the elastic range, while in cross-section M-M it goes into the elastic-plastic range. On the basis of the numerical analyses carried out, the values of critical and ultimate loads for all the analysed variants are presented in Table 2. Critical load values were determined on the basis of LBA analysis and ultimate load values were determined on the basis of GMNA analysis. In the last column of the table, the increase in load resulting from supercritical load capacity (Class 4 sections with a wall thicknesses of 1 mm and 1.5 mm) or the decrease in ultimate load in relation to critical load (Class 2 sections with a wall thickness of 4 mm) are presented. For a 2 mm thick wall, although it is a Class 4 section, that by definition is sensitive to local loss of stability, it can be noted that an increase in load due to supercritical resistance occurs only for model t2/e29. However, in models t2/103.92 and t2/150, the ultimate load in relation to the critical load decreases. Such results are certainly influenced by the type of analyses used to determine the critical and ultimate loads respectively. The critical load was determined on the basis of linear analysis (LBA), which assumes both linear material characteristics and the theory of small deflections [24]. The buckling mode obtained on the basis of LBA analysis is a local form of instability; therefore, it relates to the stability of the cross-section, and the eccentricity value in the joint does not affect the value of the critical load obtained. It is confirmed by similar values of critical load obtained for all model variants. The ultimate load was obtained on the basis of nonlinear GMNA analysis based on nonlinear elasto-plastic material model and also incorporating large deflections.

**Figure 13:**
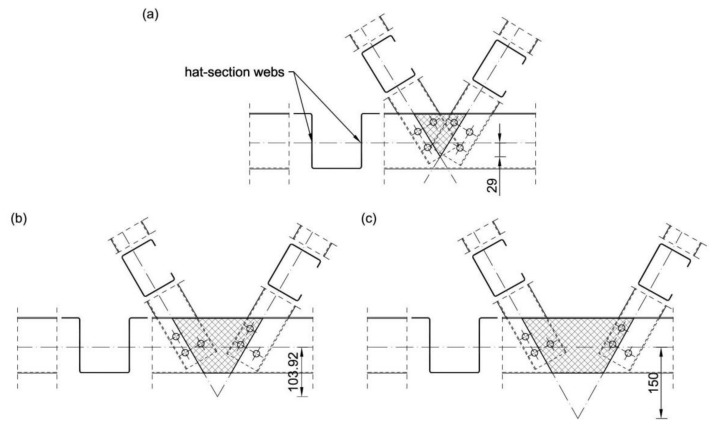
Stiffened zones of the hat-section webs for the eccentricity values: (**a**) *e_1_* = 29 mm, (**b**) *e_2_* = 103.92 mm, (**c**) *e_3_* = 150 mm.

Based on the above results, it was found that the walls of the hat sections with a thickness of 1 mm and 1.5 mm in the analysed joint area do not lose local stability despite being class 4. It should be noted that according to the theory of elastic plates, class 4 for internal elements is defined for an infinitely long panel supported at two edges [25]. However, in the analysed cases, the effect of local stiffness of the compressed and bent web plate through the walls of the diagonal channels changes the stiffness of the web plate in the joint area. Figure 13 shows the shapes of the stiffened web zones of the compressed chord web. This shape depends on the value of the eccentricity. In the case of a 2 mm-thick wall and eccentricities *e*_2_ = 103.92 mm and *e*_3_ = 150 mm, the wall in the joint area loses local stability. This indicates a smaller influence of the diagonals in the analysed joint on the web stiffening of the chord made of hat sections.

## 5. Comparison of Eurocode Calculations with the Results of Numerical Analyses

For comparison purposes, calculations of the cross-section utilization in three selected cross-sections (Figure 1) for the eccentricity value *e* = 103.92 mm were performed. These calculations were carried out in accordance with the procedures comprised in Eurocode [2] and shown in [5]. Similarly to the numerical analyses presented in point 4, the cross-section utilization was determined for wall thicknesses of 1.0, 1.5, 2.0 and 4.0 mm, including selected load cases consistent with the load history used during numerical analyses. Due to the slenderness of the walls with a thickness of 1.0, 1.5 and 2 mm, they were treated as class 4 sections. The results of the calculations are presented in Table 3.

When comparing the analytical calculations with the results of numerical analyses, it can be noticed that in all variants the value of the ultimate load (Table 2) determined numerically is greater than the value of the truss loads causing to exceed the maximum (100%) use of the cross-section in the joint (according to the assumptions of the research hypothesis). In the example of model t1.5/e103.92: *H_u_* = 65.23 kN; *P_u_* = 32.69 kN are greater than *H* = 40 kN; *P* = 20 kN. Although the results obtained from numerical study concerning wall thickness equal to 1 and 1.5 mm clearly show that the local stiffening of a hat-section’s webs by channel profile lead to the destruction (occurring in the M-M cross-section as shown in Figure 12) that occurs outside the joint. Moreover, approximate analytical calculations show that the cross-section in the joint is a critical cross-section.

## 6. Conclusions

The numerical studies presented in this paper were based on experimental studies [5] that allowed for validation of the numerical model and made it possible to study the behaviour of joints with positive eccentricity in truss girders made of cold-formed open-cross sections, taking into account different eccentricities and different wall thicknesses. The results show that:

1. The influence of local stiffening of the compressed and bent plate of the hat-section web by the walls of the diagonal channel-section was confirmed to increase the stiffness of the hat-section web walls located in the area between the diagonals. This influence is particularly noticeable in thin plate sheets with a slenderness of λ > 70,
2. For the hat-section web with a slenderness of λ < 25, no influence of local stiffening on the increase in the web walls’ stiffness was observed,
3. For the hat-section web with intermediate slenderness 25 < λ < 70, a partial influence of local stiffening on the increase in web stiffness was noticeable,
4. The walls of the hat cross-section in the joint area, due to the stiffening role of the diagonals, can be treated as walls insensitive to the local loss of stability.

The mentioned above research results could be applied in standard regulations.

## Figures and Tables

**Table 1:** Summary of the performed variants of numerical analyses (*e*_1_, *e*_2_, *e*_3_–eccentricity value).

Profile Wall Thickness	*e*_1_ = 29.0 mm	*e*_2_ = 103.29 mm	*e*_3_ = 150.0 mm
1.0 mm	t1/e29	t1/e103.92	t1/e150
1.5 mm	t1.5/e29	t1.5/e103.92	t1.5/e150
2.0 mm	t2/e29	t2/103.92	t2/e150
4.0 mm	t4/e29	t4/103.92	t4/e150

**Table 2:** Comparison of critical and ultimate load values for each numerical analysis model.

		Critical Load (LBA Analysis)		Ultimate Load (GMNA Analysis)		Load Increase (+)/ Decrease (−)	
Wall Thickness [mm]	Eccentricity Value [mm]	P_cr_ [kN]	H_cr_ [kN]	P_u_ [kN]	H_u_ [kN]	P [%]	H [%]
1.0	29	7.57	15.12	17.05	34.02	125.3	125.0
	103.92	7.62	15.22	16.2	32.33	112.6	112.4
	150	7.57	15.12	15.09	30.11	99.4	99.2
1.5	29	24.71	49.37	33.47	66.57	35.4	34.8
	103.92	24.87	49.69	32.69	65.23	31.5	31.3
	150	24.67	49.30	30.60	61.06	24.0	23.9
2.0	29	57.81	115.50	58.39	116.53	1.0	0.9
	103.92	58.14	116.17	55.62	111.23	−4.3	−4.3
	150	57.69	115.26	50.53	101.06	−12.4	−12.3
4.0	29	318.23	636.30	145.17	289.72	−54.4	−54.5
	103.92	306.38	612.60	133.13	265.71	−56.5	−56.6
	150	297.08	594.00	122.67	244.83	−58.7	−58.8

**Table 3:** Compression and bent cross-section utilization depending on wall thickness for *e* = 103.92 mm.

Load Value		Cross-Section Number	Compression and Bent Cross-Section Utilization -Depending on Wall Thickness [%]			
H [kN]	P [kN]	(Figure 1)	1.0 mm	1.5 mm	2.0 mm	4.0 mm
20	10	1-1	97.1	39.4	22.0	7.2
		2-2	121.6	49.4	27.6	9.0
		3-3	106.3	43.3	24.2	7.6
40	20	1-1	193.9	78.7	44.0	14.3
		2-2	243.0	100.52	55.2	17.9
		3-3	206.2	83.9	47.0	14.8
80	40	1-1	-	157.0	87.9	28.6
		2-2	-	219.8	123.0	40.1
		3-3	-	167.7	93.8	29.5
100	50	1-1	-	-	110.2	35.8
		2-2	-	-	153.7	50.0
		3-3	-	-	117.1	36.9
150	75	1-1	-	-	-	53.7
		2-2	-	-	-	75.1
		3-3	-	-	-	55.3
250	125	1-1	-	-	-	89.6
		2-2	-	-	-	125.1
		3-3	-	-	-	92.1

## Data Availability

Not applicable.

## Appendix A

Details of the performed numerical analysis.
